# The increased drift of steep focusing surface gravity waves

**DOI:** 10.1017/jfm.2026.11549

**Published:** 2026-05-18

**Authors:** Aidan Blaser, Luc Lenain, Nick Pizzo

**Affiliations:** 1 Scripps Institution of Oceanography, University of California, San Diego, La Jolla, CA 92037, USA; 2 Graduate School of Oceanography, University of Rhode Island, Narragansett, RI 02882, USA

**Keywords:** surface gravity waves, air/sea interactions, ocean processes

## Abstract

Irrotational monochromatic surface gravity waves possess a mean Lagrangian drift which transports mass and enhances mixing in the upper ocean. In the ocean, where many surface waves are present, it is commonly assumed that the mean Lagrangian drift can be computed independently for each wave component and summed. Here we show, using laboratory measurements and fully nonlinear simulations of two-dimensional steep focusing wave packets, that this assumption underpredicts the average transport in regions of wave focusing by up to 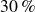 . To explain these enhancements, we derive a new exact method for constraining the local mean Lagrangian drift in general flows by working in the Lagrangian reference frame. From this method, we derive an expression for the local mean Lagrangian drift in deep-water narrow-banded wave fields governed by the nonlinear Schrödinger equation (NLSE) that predicts near-surface enhancements when waves focus and steepen. The theoretical predictions of the local transport agree with the laboratory measurements, particularly for smaller bandwidth packets where the NLSE approximation is most valid. These findings highlight that it is the local steepness of the wave field, not just the sum of the steepnesses of the linear (non-interacting) wave components, which sets the strength of these enhancements.

## Graphical abstract

**Figure:**
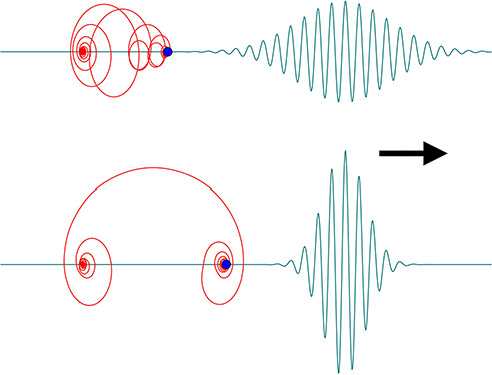

## 1. Introduction

Irrotational surface gravity waves affect the transport of mass in the ocean through their mean Lagrangian drift (van den Bremer & Breivik 2018). For steady monochromatic plane waves, this drift is horizontally uniform and increases with wave steepness (Stokes 1847). Ocean waves are neither steady nor monochromatic, and yet in most cases it is assumed that the total mean Lagrangian drift can be computed by treating the sea surface as a linear sum of non-interacting monochromatic plane waves (e.g. Kenyon 1969). In this paper we show that this assumption significantly underpredicts the near-surface mean Lagrangian drift when the surface becomes locally steep.

The mean Lagrangian drift impacts upper-ocean processes across spatio-temporal scales greater than those of individual waves, making its accurate estimation crucial to a number of applications. For example, this drift directly influences the transport and dispersal of buoyant marine debris, such as plankton, plastics and oil spills (van Sebille *et al.* 2020; Deike 2022). It is also widely understood that this vertically sheared Lagrangian-mean flow interacts with the background vorticity field to tilt and stretch vortices, producing horizontal overturning cells indicative of Langmuir circulation (Craik & Leibovich 1976; Leibovich 1983). These overturning cells help mix the upper ocean, and many studies emphasise the need to parameterise these effects in large-scale models (e.g. Belcher *et al.* 2012). Additionally, the generation of waves by wind is modified by the drift magnitude (Seitz, Freilich & Pizzo 2026). Any enhancements to the mean Lagrangian drift, especially in steep wave fields where its magnitude is largest, can therefore have a profound effect on these upper-ocean processes.

The impetus for this work came from a series of laboratory experiments (Lenain, Pizzo & Melville 2019; Sinnis *et al.* 2021) which measured the total Lagrangian displacement of surface particles induced by breaking and non-breaking wave packets. These packets consisted of multiple wave components which were tuned to constructively interfere or focus at a prescribed location and time via dispersion. Wave breaking was found to greatly increase the Lagrangian transport, with the enhancements strongly dependent on each particle’s distance from the breaking location. Interestingly, a similar, albeit weaker, spatial dependence was observed for steep non-breaking packets, with the largest enhancements occurring within the focusing region where the packet was most steep. This result was unexpected, since when viewed as a sum of linear monochromatic plane waves, the only differences between a focused and unfocused packet are relative phase shifts between wave components. If the total mean Lagrangian drift could be obtained by summing the individual drifts of each wave independently of the others, the relative phase shifts should be irrelevant. Thus, one should expect both the total drift and net transport to be spatially constant and independent of packet focusing.

To supplement the limited laboratory data, we present numerical simulations of surface Lagrangian particle trajectories in equivalently defined packets using a fully nonlinear potential flow solver (Longuet-Higgins & Cokelet 1976; Dold 1992). With a high spatial particle density, these simulations can better capture the spatial dependence of the surface Lagrangian transport. Repeating these simulations over a wide parameter space of steepness and bandwidth parameters reveals that the surface transport of particles averaged over the focusing region can exceed the spatially invariant predictions of linear theory by up to 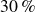 . Some individual particles can even be transported up to twice this prediction, all without any wave breaking.

It should be clear that one cannot predict local enhancements to the mean Lagrangian drift without a local theory to explain it. By working in the Lagrangian reference frame, we derive a new exact technique for constraining the local mean Lagrangian drift of general wavy flows through the local mean pseudomomentum. This result is similar to the circulation theorem in generalised Lagrangian-mean (GLM) theory (Andrews & McIntyre 1978) but presented in a fully Lagrangian framework. Leveraging this new method, we derive an expression for the local mean Lagrangian drift in two-dimensional, deep-water and narrow-banded wave packets governed by the nonlinear Schrödinger equation (NLSE) (Zakharov 1968; Chu & Mei 1970). This result complements previous estimates of the mean Lagrangian drift in wave packets derived via Eulerian frameworks (van den Bremer & Taylor 2016; Deike, Pizzo & Melville 2017; Carter, Curtis & Kalisch 2020; Li & Li 2021), which allow for greater generality in directionality, depth and bandwidth, but are valid only to leading order in wave steepness. In this derivation, we retain the effects of finite bandwidth and steepness beyond leading order, and show that their combined influence enhances the near-surface mean Lagrangian drift during wave focusing and steepening. We then use this analytical expression to estimate local enhancements to the mean Lagrangian transport in the simulations, finding good agreement, especially for lower bandwidths where the NLSE approximation is most valid.

This paper is organised as follows, in § 2, we introduce the equations of motion in the Lagrangian reference frame and derive a novel method to compute the local mean Lagrangian drift for general wavy flows. In § 3, we define the focusing wave packets used, and numerically simulate their surface particle trajectories, comparing the results with laboratory data. In § 4, we derive the Lagrangian particle trajectories in narrow-banded waves and compute a higher-order expression for the local mean Lagrangian drift, testing this theory against the simulations. In § 5, we discuss the implications of these results in broader geophysical contexts.

## 2. The mean Lagrangian drift of waves

There are two natural coordinate systems for representing fluid motion within surface gravity waves: the Eulerian and the Lagrangian. The Eulerian frame solves for the fluid velocity as a function of fixed physical space and time and is mathematically appealing due to the fact that, for two-dimensional irrotational and incompressible flow, the fluid velocity is analytic in the interior. This means that the entirety of the flow is determined by its behaviour at the boundaries (Luke 1967). The fluid interior is governed by the linear Laplace equation
(2.1)

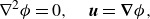

for the velocity potential 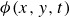 , whose spatial gradient is the Eulerian fluid velocity 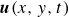 . Despite the equation of motion being linear, the problem is made considerably more difficult due to the nonlinear boundary conditions (see, for example, § 3.1 of Phillips 1977)
(2.2)

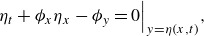



(2.3)

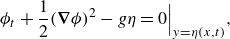




where 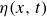 is the surface elevation, an introduced independent variable not known *a priori*, and 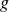 is the acceleration due to gravity, which in our notation points in the 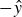 direction, with subscripts indicating partial derivatives. While it is common to evaluate these boundary conditions by expanding in a Taylor series about the still water level 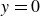 , this introduces infinitely many nonlinear terms which are in practice truncated by invoking some small parameter which is typically related to the surface wave slope. To compute the physical trajectories of fluid particles as functions of their initial positions and time, one must then integrate the coupled pathline equations
(2.4)

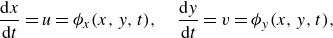

holding particles fixed. Solving (2.4) yields Lagrangian particle trajectories correct to the same level of accuracy as the Eulerian velocity field. However, in practice, analytical solutions quickly become intractable as one must account for changing particle locations in the velocity field. If the desired result is to compute the mean Lagrangian motion of particles, it is much more natural to work directly in the Lagrangian reference frame, where the physical particle trajectories 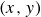 are explicitly solved for as functions of general labelling coordinates 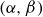 and time 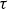 , which we distinguish from the usual notation 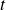 to emphasise that a partial derivative with respect to 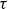 holds particle labels fixed (i.e. equivalent to the material derivative 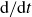 in the Eulerian frame). One can view these physical trajectories as a time-dependent mapping from a certain ‘label space’ to physical space with a corresponding Jacobian determinant
(2.5)

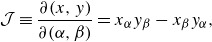

whose value determines how infinitesimal areas are scaled by the nonlinear mapping. Since incompressible flow requires that a small collection of particles 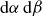 enclose the same physical area 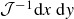 as the flow evolves, we see that 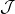 must be everywhere time independent and non-zero,
(2.6)

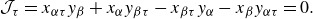

The particular choice of labelling particles must not affect the dynamics and thus represents an important gauge freedom in fluid mechanics (Salmon 2020). For simplicity we hereinafter choose to work with a labelling gauge such that 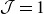 , so that areas in label space equal areas in physical space. While we can still define a velocity potential in the Lagrangian frame for irrotational flow, the generally nonlinear mapping between physical and label space implies that the form of the Laplacian operator is more complicated in label space as these maps are not generally harmonic. Instead, we turn to the full Euler equations which in the Lagrangian frame are written as (Lamb 1932, Art. 15)
(2.7)

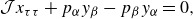



(2.8)

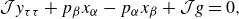

where 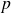 is the fluid pressure. Note that while the material acceleration is greatly simplified in the Lagrangian frame, the pressure gradient force is no longer represented by a simple linear operator. In practice any Eulerian quantity or operator can be converted to the Lagrangian frame through the Jacobian. For example, the vorticity of the fluid can be converted to the Lagrangian frame via the following steps:
(2.9)

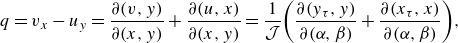

where 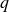 is conserved on particles (i.e. 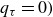 for two-dimensional inviscid flow, which can be seen by eliminating 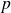 between the two Euler equations. The strict condition of irrotational flow thus imposes the following constraint on the fluid trajectories:
(2.10)

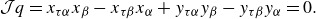

To close the system, we impose the following boundary conditions: first, that the pressure vanishes up to a constant at the free surface which we label by our choice as 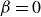



(2.11)

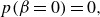

and second, that the vertical velocity vanishes approaching the bottom at infinite depth
(2.12)

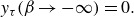




Note that, while we have necessarily abandoned the simplicity of Laplace’s equation for more complicated nonlinear equations of motion (2.7)–(2.8), what we have gained from this approach is having simple boundary conditions without potentially infinite nonlinear terms which necessitate small-amplitude approximations. In addition, as vorticity is conserved on particles, adding arbitrary vorticity to particles is straightforward in the Lagrangian frame as opposed to in the Eulerian frame where Laplace’s equation would have to be replaced with the full nonlinear Euler equations alongside the nonlinear boundary conditions.

### 2.1. Mean Lagrangian drift of general flows

While directly solving the Euler (2.7)–(2.8) subject to 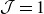 , 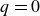 and the boundary conditions (2.11)–(2.12) will yield particle trajectories that explicitly contain the mean Lagrangian drift, this offers little physical insight into its origin. Previous studies connected the mean Lagrangian drift, or equivalently the mean Lagrangian momentum density, to other physical quantities such as vorticity and energy (Pizzo *et al.* 2023; Blaser *et al.* 2024), but these results necessarily assumed waves that were steady and monochromatic. In this section, we introduce a new method of constraining the mean Lagrangian drift for completely general flows. To do so, we start by considering the circulation of a material loop 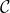 , which is defined as
(2.13)

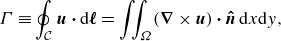

with the last relation due to Stokes’ theorem for the area 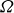 enclosed by the contour where 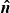 is the unit outward normal. We simplify here to two-dimensional flow, but the following results may be readily extended to three dimensions (Salmon 1988). Just as with the vorticity, we can rewrite the circulation in Lagrangian coordinates via the chain rule
(2.14)

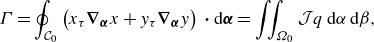

where 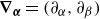 is the gradient operator in label space. The contour 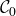 is now a contour in label space and is therefore fixed in time by definition; the same goes for the enclosed area 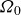 . If we decompose the Lagrangian trajectories into deviations from a quiescent reference state 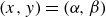



(2.15)

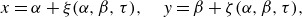

so that 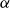 and 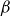 can be seen as ‘horizontal’ and ‘vertical’ labels respectively, we can rewrite the circulation as
(2.16)

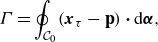

where 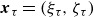 is the Lagrangian velocity, and 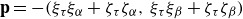 is identified as the Lagrangian pseudomomentum. While its form looks identical to pseudomomentum as defined in GLM theory (Andrews & McIntyre 1978; Bühler 2014), they are still distinct since the displacement vector in GLM is a function of the Lagrangian-mean trajectory, not Lagrangian particle labels. For irrotational flows where 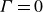 for all closed loops, (2.16) implies that the label space curl of the velocity must be everywhere equal to the label space curl of the pseudomomentum
(2.17)

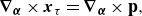

analogous to the celebrated result in GLM (Bühler 2014, Ch. 10). Since what we are interested in is the mean component of the velocity, we can take an average of (2.17) to get
(2.18)

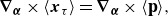

where the angle brackets represent any general averaging operator in the Lagrangian frame that commutes with the curl in label space, such as a time mean or convolutional average. It is worth pausing here for a moment to unpack this result, which states that, for irrotational flow, the curl of the mean Lagrangian drift is exactly set by the curl of the mean pseudomomentum so that any modification to one immediately affects the other. Viewing the mean Lagrangian drift in relation to the dynamic mean pseudomomentum highlights its role as not simply a passive byproduct of the waves, but as a dynamic mean flow in its own right. This view will be especially helpful when we turn to the mean Lagrangian drift of narrow-banded wave packets. However, for completeness, we will use this new general framework to compute the mean Lagrangian drift for linear waves in the following subsections.

### 2.2. Monochromatic waves

We start with the classical example of a linear deep-water monochromatic wave with wavenumber 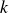 and constant amplitude 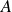 where the non-dimensional steepness 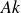 is assumed to be small. Following the method of (Salmon 2020, § 1), we assume a wavelike solution for 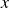 and 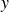 after expanding about a hydrostatic state of rest ( 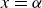 , 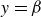 , 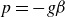 )
(2.19)

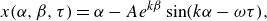



(2.20)

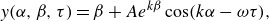



(2.21)

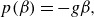




where 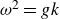 is the linear deep-water dispersion relation determined by substituting (2.19)–(2.21) into the Euler (2.7)–(2.8). These simple circular trajectories are in fact exact solutions to the Euler equations known as Gerstner (1802) waves. However, these waves are not irrotational, which can be seen by computing their vorticity using (2.9). From (2.18), we see that irrotational flow requires that the curl of the mean Lagrangian drift be equal to the curl of the mean pseudomomentum. Computing the pseudomomentum of (2.19)–(2.20) yields only a horizontal component
(2.22)

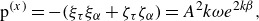

that varies only with depth. Taking the mean to be a long time average following a fixed particle, from (2.18) we require
(2.23)

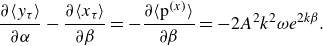

On physical grounds we can assume there is no mean vertical velocity, so that the solution to (2.23) is
(2.24)

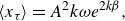

where the arbitrary constant of integration can be removed in the frame where the velocity of fluid at depth vanishes. This the classical Stokes drift. We reproduce it here as an example of our general method but also because it shows how the second-order mean flow is constrained by first-order orbital motion, due to the pseudomomentum being a quadratic quantity. This carries to higher-order corrections as well; since the particle displacements in surface gravity wave fields 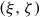 are always first-order quantities or higher, one needs only to constrain trajectories valid to order 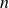 to constrain the drift to order 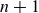 .

### 2.3. Multiple waves – lowest-order theory

Following Pierson (1961), if we instead consider a discrete spectrum of 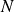 deep-water plane waves travelling in the same direction, to first order we have
(2.25)

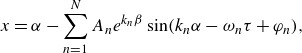



(2.26)

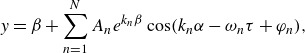




where 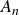 , 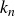 , 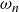 and 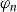 are the amplitude, wavenumber, frequency and initial phase of each wave component, respectively. It is assumed that each wave’s steepness 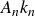 is small and, importantly for this analysis, constant. The horizontal component of the pseudomomentum is given by products of sums, but taking the mean to be a long time average following a fixed particle, we have
(2.27)

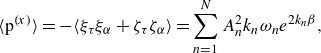

where any cross-terms vanish in the time mean due to to the fact that 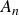 , 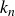 , 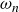 and 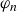 are constant. From (2.18),
(2.28)

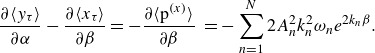

Once again, the only physically valid solution is balanced by 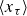 , so that
(2.29)

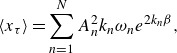

where the constant of integration vanishes in the frame where the fluid interior is at rest. We see that according to lowest-order theory, the total mean Lagrangian drift for a wave field is a simple linear sum of the individual drifts of each wave component. While the full second-order particle trajectory solutions contain bounded second-order harmonics (Pierson 1961; Nouguier, Chapron & Guérin 2015), which can be interpreted as local fluctuations to the mean Lagrangian drift, these terms are fully oscillatory and do not contribute to the long time transport regardless of how the initial phases 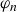 are tuned. We will henceforth refer to this as the linear theory, on account of its additive property, although this should not be confused with linearity with respect to wave steepness. From this theory, the effect of local steepness fluctuations to the mean Lagrangian drift is symmetric; any local increases during constructive interference are cancelled by local decreases during destructive interference. We would therefore expect the total transport of a passing wave packet, expressed as a sum of plane waves, to be similarly invariant to local wave focusing. In the following section, we investigate the Lagrangian transport of focusing wave packets, presenting numerically simulated particle trajectories alongside laboratory data.

## 3. Lagrangian transport due to focusing wave packets

We now narrow our scope to spatially compact focusing wave packets. First, we define these packets and provide a linear prediction of their induced surface Lagrangian transport. Next, we introduce the fully nonlinear solver used to simulate the Lagrangian trajectories of surface particles, and show the results of these simulations for a range of packet parameter space. Finally, we compare the results of the simulations and laboratory experiments against the predictions of linear theory.

### 3.1. Packet initialisation

We define our packets as in Rapp & Melville (1990), Drazen, Melville & Lenain (2008) and Sinnis *et al.* (2021) to focus according to linear theory at a prescribed space and time
(3.1)

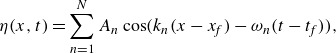

where 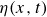 is the Eulerian free surface displacement, 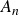 is the amplitude of each discrete wave, 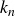 and 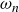 represent the respective wavenumber and frequency of each component, both positive as all wave components travel to the right, and 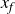 and 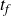 denote the focusing location and time respectively according to linear theory. We consider a uniformly distributed spectrum in frequency space, so that our frequencies can be expressed as
(3.2)

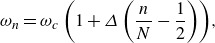

where 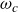 is the central frequency (so that 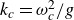 is the central wavenumber) and 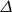 is the non-dimensional bandwidth equivalent to 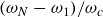 which sets the time and space scales of the focusing event and must be less than 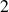 to ensure positive frequencies. In addition, as the slope of waves is an indicator of their nonlinearity, we wish to define the amplitudes 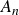 such that at focusing, the linear prediction of the maximum slope equals some prescribed value 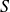 . Therefore, we define
(3.3)

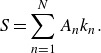




Thus, given the linear deep-water dispersion relationship 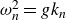 , we can determine the values of 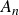 , assuming the slope of each mode is equal following Drazen *et al.* (2008) (i.e. 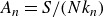 ). Placed in this formulation, the wave packets we consider are primarily functions of two independent variables, 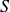 and 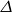 , which will be used as our parameter space.

The linear prediction of the surface mean Lagrangian drift is given by a simple sum of the lowest-order contributions (2.29)
(3.4)

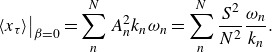




Based solely on (3.4), the surface mean Lagrangian drift scales as 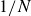 , which implies that a packet with more waves experiences less drift, despite the fact that 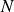 simply represents the spectral resolution of the packet, whose form converges as 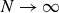 . This is due to the fact that the temporal periodicity of (3.1) is given by
(3.5)

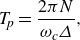

so that as 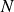 increases, the time between subsequent packets also increases. Since what we are after is not the mean Lagrangian drift itself, which according to (2.29) is the same for all particles at all times since it treats the packet as a sum of monochromatic plane waves, we instead compute the total linear surface Lagrangian transport 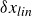 after a single packet has passed. This is done by integrating (3.4) in time over the temporal periodicity of the packet (3.5)
(3.6)

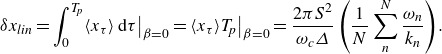




We see that the linear prediction of the total surface Lagrangian transport should scale with 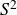 as one should expect for the lowest-order theory. The transport scaling inversely with 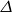 should also be expected as the packet width in physical space is inversely proportional to its width in wavenumber space (Sinnis *et al.* 2021). The last term represents a spectrally weighted phase speed. For 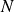 large, (3.6) can be approximated in closed form from (3.2) as
(3.7)

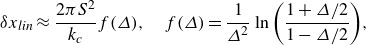

where 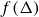 represents the linear bandwidth dependence on the total transport, found by approximating the sum in (3.6) as an integral. While this full expression is slightly more complicated than the heuristic argument given above, 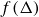 is well approximated by 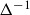 when 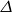 is small.

### 3.2. Numerical simulations of Lagrangian trajectories

To simulate the Lagrangian trajectories of surface particles within these packets, we employ a fully nonlinear mixed Eulerian–Lagrangian potential flow solver (Dold 1992). Originally developed by Longuet-Higgins & Cokelet (1976), this method takes advantage of the fact that, at a fixed time, the Eulerian and Lagrangian velocities are equal since a particle occupies a single fixed location at a fixed time. Because solutions to Laplace’s (2.1) are uniquely determined by the boundary conditions, only the surface needs to be simulated, assuming a constant or infinite depth and a periodic domain. By initialising Lagrangian particles with initial positions 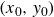 and velocity potential 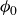 , this solver computes the gradient of 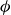 at the surface given its value via Cauchy’s integral theorem at each time step. This allows for the particle positions 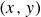 to evolve via the pathline (2.4), with the velocity potential evolving according to Bernoulli’s equation at the free surface (2.3). Because Lagrangian particles naturally cluster at wave crests where the spatial curvature is strongest, the resolution of this method is naturally adaptive, and numerous studies (Dommermuth *et al.* 1988; Skyner 1996) have validated the accuracy and validity of this numerical method.

For our simulations, we chose a central wave frequency of 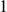 Hz, so that 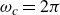 rad s^−1^ and 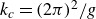 rad m^−1^. The domain length was chosen to be 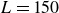 m, long enough so that the entire packet could fully pass over a large enough collection of particles to obtain an unambiguous measure of total Lagrangian transport before any signal wrapped around due to the periodicity of the domain. To fully resolve the free surface, we systematically increased the number of Lagrangian particles used until convergence was reached at 2048 particles, or around 20 per central wavelength. The depth of the water is taken to be infinitely deep, and the packets were initialised to start 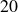 m away from the prescribed focusing location so that there were sufficient particles within the focusing region that both started and ended at rest. We defined the linear prediction of the focusing time as 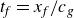 , where 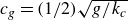 is the central group velocity according to linear theory. Lastly, we chose to use 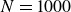 wave modes so that the spectral resolution is sufficiently high to converge the physical shape of the packet.

The procedure for simulating these packets is as follows. First, we initialise the horizontal positions 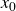 to be evenly spaced along the domain. Then, using (3.1), the vertical initial positions 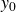 are found for prescribed values of 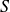 and 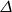 . To ensure only one packet is used and that the domain is totally periodic, a windowing function is applied to 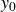 with minimal energy loss (less than one part in 100). The initial velocity potential is found according to linear theory by performing a Fourier transform on the windowed 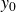 , multiplying each Fourier amplitude by 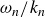 , and performing an inverse transform. The simulations were repeated for a parameter space spanning 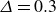 , incremented by 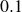 until 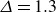 , and 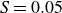 , incremented by 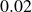 until the packet broke, which agreed well with the results of Pizzo & Melville (2019); see also Pizzo *et al.* (2021), who numerically investigated the breaking threshold 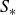 of these same packets as a function of bandwidth. To improve parameter space resolution near 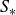 , we ran additional simulations near this threshold.

**Figure 1.**
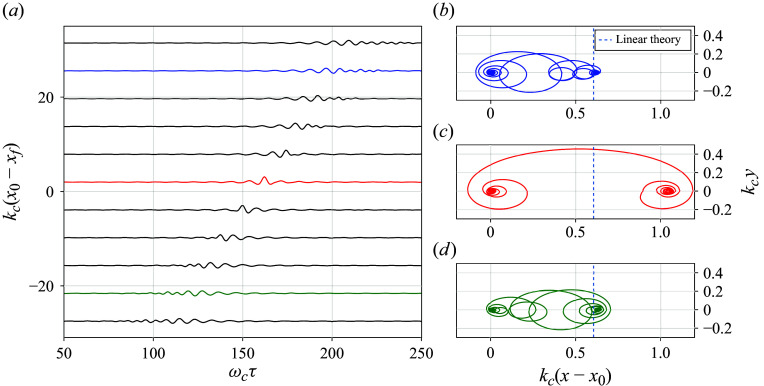
Surface particle trajectories in a focusing wave packet with 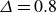 and 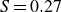 . In panel 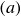 , the vertical elevation of individual fluid particles is plotted as a function of time. Each curve represents a different particle, labelled by its initial horizontal distance from the focusing region 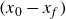 , normalised by the central wavenumber 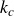 , as shown on the vertical axis. The coloured lines represent particles downstream of focusing (blue), at focusing (red) and upstream of focusing (green). Likewise, on the right, panels (*b*,*c*,*d*) show the physical particle trajectories of these downstream, at focusing, and upstream particles respectively, normalised by 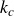 . Note that the total transport during focusing (red) is much greater than that away from focusing, contrary to linear theory (3.6) (dashed line) which states that all particles should experience the same transport.

Figure 1 shows a typical output of the surface particle trajectories during one such focusing event with bandwidth 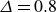 and linear prediction of maximum slope at focusing 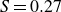 . For particles far downstream and upstream of focusing, represented by the blue and green curves respectively, their trajectories evolve gradually as the packet passes over. Their measured total transport 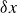 , represented by the difference from their final and initial positions, mostly follows linear theory (3.6). In contrast, for the particles at or near the focusing location, highlighted by the red curve, the transport occurs in one short burst as the focused packet passes over, well surpassing the predictions of linear theory and violating the supposed spatial invariance of the transport. For each particle, the total Lagrangian transport 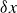 is computed by taking the horizontal position averaged over the final two seconds of the simulation, roughly two central wave periods, and subtracting from it the particle’s fixed initial position 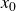 . Both here and for the rest of this paper, we only show results for particles that began and ended at rest (i.e. they experienced the full packet passing) so that total transport 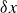 is unambiguous.

**Figure 2.**
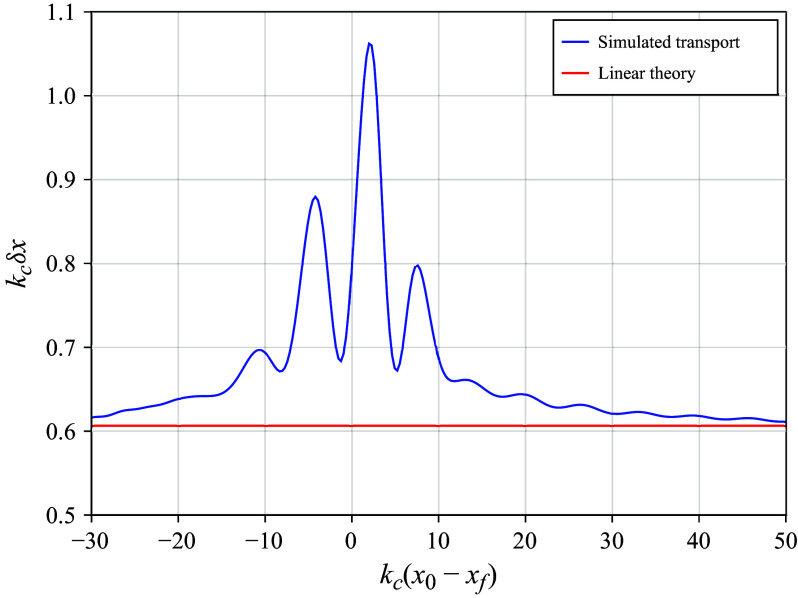
The total Lagrangian transport 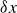 of surface particles as a function of their initial distance from the linear prediction of maximum focusing 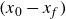 , normalised by the central wavenumber 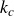 for the same simulation as in figure 1, 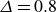 and 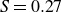 . The normalised linear prediction of the total transport 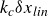 (3.6), constant in space, is shown in red.

Figure 2 shows the computed total surface Lagrangian transport 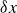 as a function of its initial position relative to the linear focusing prediction, both normalised by the central wavenumber 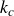 for the same simulation as in figure 1. Plotted also is 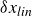 , normalised by 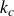 , constant for each particle. From figure 2, we see a strong spatial dependence of the total Lagrangian transport, with a maximum transport 75 % higher than linear theory predicts. At larger values of 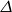 , where the physical packet width at focusing is smaller, the maximum transport was even found to be up to double that of linear theory. These are surprising results as it might be expected that any higher-order corrections to linear theory would be necessarily small. Here, we show that these corrections are comparable in magnitude to the linear prediction and exhibit a strong spatial dependence. In addition to the general increase around the focusing location, all simulations have oscillations in their transport curve near focusing with a spatial periodicity that matches the wavelength of the central wave. To compare these results with laboratory experiments, we also introduce a measure of the mean surface transport over the focusing region following Sinnis *et al.* (2021),
(3.8)

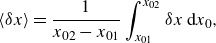

where in our study we define 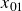 and 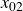 as the first and last points, respectively, where the deviation of the transport from linear theory 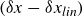 exceeds 10 % of its maximum value. In this case the mean transport is 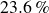 higher than linear theory. Although this particular definition of the mean transport is by no means unique, it was chosen to best match the approach used in Sinnis *et al.* (2021).

While these simulations provide the first detailed account of the increased transport of steep non-breaking focusing wave packets, this study was motivated by earlier laboratory experiments (Lenain *et al.* 2019; Sinnis *et al.* 2021). These wave tank experiments measured the spatially varying surface transport in primarily breaking focusing wave packets described by (3.1), with several steep non-breaking cases included for comparison. They found that wave breaking produces a large local increase to the surface transport. Wave breaking, in this case, breaks both the translational symmetry of the system and the transport in an obvious way. However, this symmetry breaking is also present for non-breaking focusing waves, allowing for a spatially dependent non-breaking transport which can be seen for example in Sinnis *et al.* (2021) figure 5.

**Figure 3.**
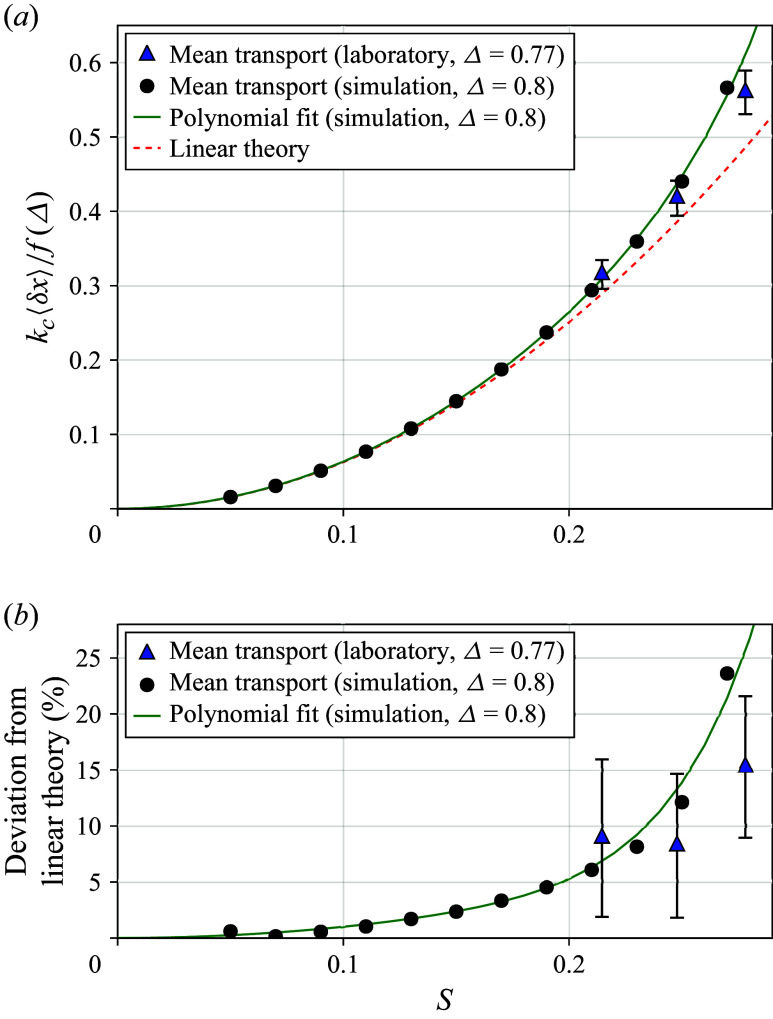
Mean surface transport 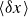 as a function of the linear prediction of maximum wave slope 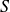 . Panel 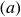 shows the mean transport normalised by the central wavenumber 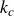 and linear bandwidth dependence 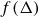 so that the prediction of linear theory (3.6) (red) collapses to a single curve for both the simulation and laboratory parameters. A polynomial fit of the discrete simulation points is shown in green. Panel 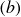 shows the same data plotted as a percentage increase from linear theory.

Figure 3 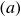 compares the mean surface transport from laboratory experiments and simulations as a function of 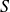 , normalised by the central wavenumber 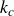 and linear bandwidth dependence 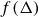 so that the linear theory (3.7) coincides for both cases. For the laboratory experiments, conducted in a finite-depth tank of mean depth 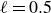 m, this requires using the full dispersion relationship 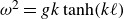 to numerically compute 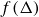 . Despite these differences, a clear trend emerges: the mean surface transport exceeds the predictions of linear theory as 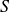 increases. To better visualise these increases, figure 3(*b*) plots the same data instead as a percentage deviation from linear theory, revealing that these enhancements are of comparable magnitude to the linear theory itself.

**Figure 4.**
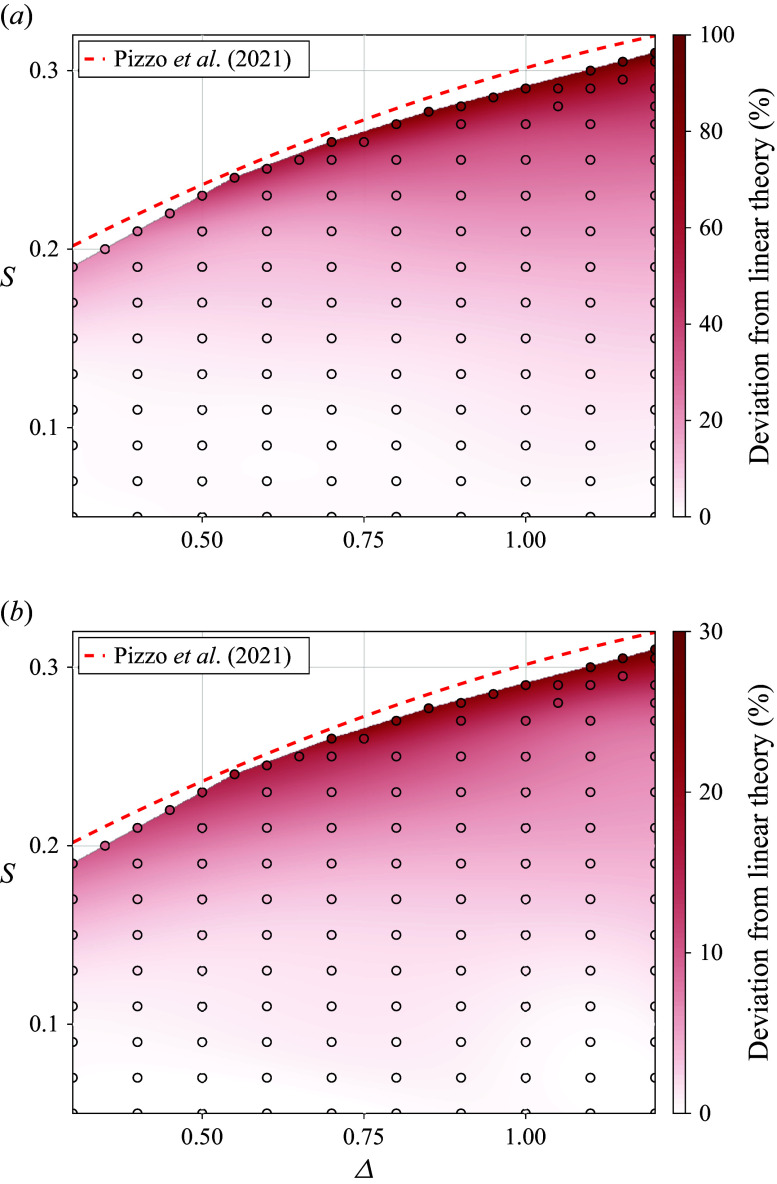
Percentage increases of the maximum 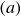 and mean 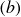 surface Lagrangian transport relative to linear theory (3.6) for numerically simulated focusing wave packets as a function of parameter space 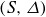 . Discrete simulation runs are shown via coloured markers, with interpolated values in between. Note the two distinct colour bar scalings for panels (*a*,*b*). The red line outlining the parameter space represents the breaking slope threshold numerically determined by Pizzo *et al.* (2021) which we found to be consistent with our simulations.

The enhancements of the maximum and mean surface transport relative to linear theory for all simulations are shown in figure 4 as discrete points, with interpolated values in between. The dashed red line indicates the breaking slope threshold 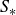 numerically determined by Pizzo *et al.* (2021) for equivalently defined deep-water packets. Individual particles, shown in figure 4 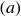 , can be transported up to twice as far as linear theory predicts, with the mean transport over the focusing region surpassing linear theory by up to 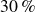 as shown in figure 4 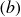 . Although the enhancements to the surface Lagrangian transport primarily scale with increasing 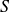 , there is also a noticeable 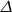 dependence close to the breaking threshold. To investigate why these spatially varying enhancements occur when waves steepen, we next turn to a theoretical derivation of the local mean Lagrangian drift in deep-water narrow-banded waves.

## 4. Wave packets in the Lagrangian frame

We begin by considering a unidirectional wave packet in infinitely deep water with a characteristic wavenumber 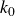 and frequency 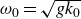 , and for simplicity normalise our units with new primed variables
(4.1)

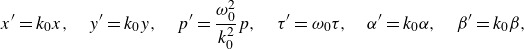

which we henceforth drop for clarity of presentation. Using 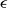 as a small steepness parameter, analogous to 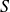 , we start with non-dimensionalised monochromatic waves
(4.2)

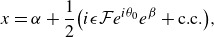



(4.3)

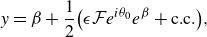



(4.4)

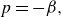




where 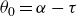 , 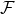 is an 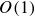 non-dimensional complex amplitude and 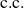 indicates the complex conjugate. At this order 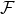 is constant and the solutions represent monochromatic plane waves identical to (2.19)–(2.21). To account for the effects of finite bandwidth, we allow for this complex amplitude to vary slowly in space and time, so that 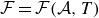 where we have introduced the new slow variables
(4.5)

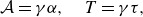




where 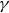 is another small parameter which is proportional to the normalised bandwidth 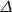 . We separate 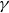 from 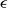 to show how finite steepness and bandwidth individually affect the solutions following the approach of van den Bremer & Taylor (2016), but assume both to be small parameters of the same asymptotic ordering so that a second-order quantity, for example, describes terms proportional to any of the following: 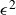 , 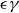 or 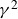 . The general procedure for computing higher-order solutions is to expand 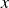 , 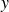 and 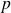 in a standard asymptotic series starting at second order
(4.6)

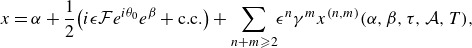



(4.7)

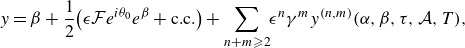



(4.8)

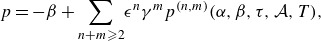




where 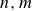 are non-negative integers. Inserting (4.6)–(4.8) into the Euler (2.7)–(2.8), the irrotational condition (2.10) and the continuity gauge choice 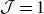 , and grouping terms by powers of 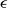 and 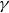 , we obtain a set of linear equations at second order. Solving them along with the relevant boundary conditions yields
(4.9)

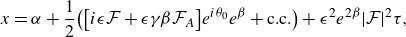



(4.10)

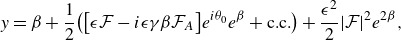



(4.11)

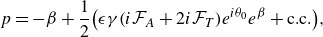




where the oscillatory motion now no longer decays purely exponentially with depth, similar to what is found in the Eulerian frame for narrow-banded packets (Yuen & Lake 1975; Pizzo & Melville 2016). Just as with monochromatic waves, a second-order mean Lagrangian drift is required to enforce irrotational flow, although here its strength is set by the local squared magnitude of the packet envelope (van den Bremer & Taylor 2016; Haney & Young 2017). The presence of the waves also raises the potential energy of the fluid, as seen in (4.10) through the Lagrangian-mean water level. The condition that pressure vanishes at the sea surface requires 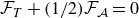 which simply means that to lowest order the envelope translates with the non-dimensional linear group velocity, which in deep water is half of the phase velocity.

As reported by Buldakov, Taylor & Taylor (2006) for monochromatic waves, directly continuing this asymptotic expansion to third order yields non-physical oscillatory terms in 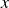 and 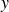 which grow secularly in time. To obtain uniformly valid particle trajectories, Clamond (2007) identified that the phase of the waves must be Doppler shifted by the mean Lagrangian drift, effectively renormalising the phase. This correction is necessary since two particles that are initially in phase (same 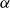 , different 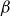 ) gradually move out of phase at long times due to the vertically sheared mean Lagrangian drift transporting one more than the other. To account for the effects of the vertically sheared mean Lagrangian drift in our system (4.9), and because our solutions must reduce to those of monochromatic waves when the envelope is constant in space, we adopt the same renormalisation of the carrier wave phase
(4.12)

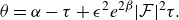




At third order, this yields the solutions
(4.13)

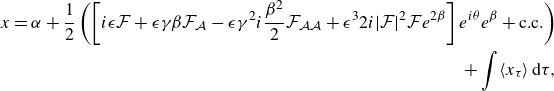



(4.14)

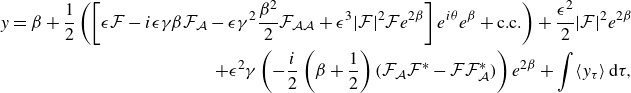



(4.15)

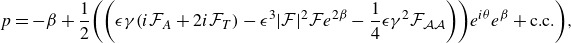




where the mean Lagrangian drift, left in general form here, is formally derived in the following subsection. It is important to note that only the second-order mean terms are required to fully constrain the third-order orbital motion. From the condition of vanishing pressure at the free surface, and following Abrashkin & Pelinovsky (2017) and Pizzo *et al.* (2023), we derive an equation governing the evolution of the wave envelope
(4.16)

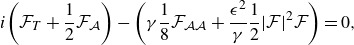

which reduces to the classical NLSE for narrow-banded irrotational waves when 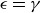 (Zakharov 1968). This (4.16) has a number of conserved quantities in time. These are, as they are commonly referred to in the literature, the linear wave energy
(4.17)

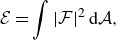

the mean wavenumber
(4.18)

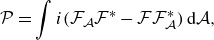

and the Hamiltonian
(4.19)

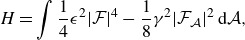

which arise via Noether’s theorem from symmetries of the NLSE action to phase shifts, spatial translation and time translation, respectively (Sulem & Sulem 1999). The conservation of 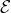 ensures that 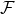 remains bounded for a finite packet, and therefore the third-order solutions (4.13)–(4.15) are free of secular amplitude growth.

From these conservation laws, it follows that the time integral of 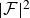 is conserved to third order. The leading-order mean Lagrangian drift (4.9) therefore predicts that the net Lagrangian displacement 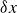 ,
(4.20)

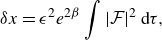

is conserved on fluid particles and independent of focusing (Deike *et al.* 2017, Appendix B). Consequently, although the NLSE accounts for both dispersion and weak nonlinearities, second-order envelope-based predictions cannot explain the localised enhancement of the total Lagrangian transport observed during focusing events, necessitating higher-order predictive theories.

### 4.1. The mean Lagrangian drift of steep narrow-banded waves

Because we enforced a scale separation between the fast orbital motion and the slow envelope evolution through the small parameter 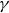 , the averaging operator 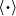 is defined as a spatial convolution over an intermediate scale – large enough to remove the fast oscillations but small enough to retain slow envelope modulations. Spatial convolutions commute with the curl, so that from (2.18) we know that the curl of the mean Lagrangian drift is exactly equal to the curl of the mean pseudomomentum. The pseudomomentum itself is computed from products of the Lagrangian particle displacements 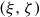 whose leading-order contributions are 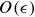 ; consequently, the fourth-order pseudomomentum is determined entirely by third-order terms. It is easy to check that the second-order mean terms in (4.9)–(4.10) only contribute to the curl of the mean pseudomomentum beginning at fifth order, so that only the oscillatory terms are required. Direct evaluation of this quantity from (4.13)–(4.14) shows that the mean Lagrangian drift must satisfy
(4.21)

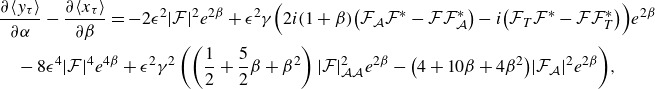




where we have used the identity
(4.22)

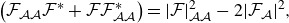

to consolidate various terms. In its current form (4.21) is underdetermined since we cannot ignore 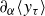 at higher orders. Although the fluid is incompressible, the mean Lagrangian velocity need not be divergence free (2.6). As pointed out by Vanneste & Young (2022), the fact that waves modify the potential energy of a fluid implies a changing centre of mass, which, when paired with the bottom boundary condition, requires a divergent Lagrangian-mean flow. We can account for this, however, in the Lagrangian-mean water level, whose value can be computed from the Jacobian (2.5) independently of the mean Lagrangian drift to fourth order from (4.13)–(4.14)
(4.23)

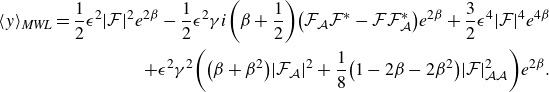




The Lagrangian-mean water level (4.23), or more aptly changes thereof, fully constrain the divergent part of the total mean Lagrangian velocity. This can be seen by taking the average of the incompressibility condition (2.6), which from (4.13), (4.14) and (4.23) imply
(4.24)

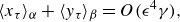

so that, to fourth order, the mean Lagrangian drift is divergence free (at higher orders products of the mean Lagrangian drift enter (4.24)). This allows us to define a streamfunction for the mean Lagrangian drift 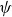 such that
(4.25)

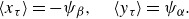




Equation (4.21) does not distinguish between the mean Lagrangian drift or the Lagrangian-mean water level, so to only constrain the drift we must account for the mean water level’s contribution to the curl, which only emerges at fourth order
(4.26)

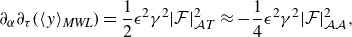

where for simplicity we have used the fact that, to lowest order, 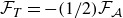 . Combining (4.21) with (4.25) therefore results in
(4.27)

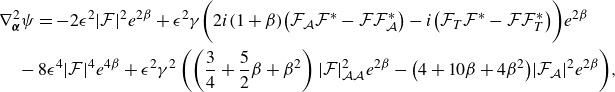

which is just the linear Poisson equation. One can interpret (4.27) as equating the vorticity of the mean Lagrangian drift to the curl of the mean pseudomomentum, which acts here as a wave-induced source of vorticity (Salmon 2020). It is important to reiterate that, despite this interpretation, the vorticity of the fluid is still exactly zero everywhere in the fluid. The monochromatic mean Lagrangian drift shows how a mean flow that is sheared in the Lagrangian reference frame can still describe perfectly irrotational flow.

To close the system, we require boundary conditions on 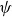 . The first one is simple: the flow vanishing at infinite depth implies 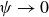 as 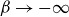 . The surface boundary condition is more subtle, as it was implicitly introduced in § 2 from the fact that the surface of the fluid is always defined by particles with 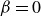 . This is the Lagrangian equivalent of the kinematic boundary condition, (2.2) in the Eulerian frame, which in plain language states that particles which start at the surface always remain at the surface. From the perspective of the mean Lagrangian drift, there can therefore be no mass flux through the surface, making it a streamline which we can without loss of generality set to 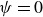 at 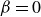 . This is not to say that there can be no mean vertical motion, only that any such motion at the surface must correspond with changes to the surface geometry, which is already set by the Lagrangian-mean water level. Slow changes in the Lagrangian-mean water level, found by taking a time derivative of (4.23), can be interpreted as a ‘vertical drift’ which several studies investigate (e.g. Vanneste & Young 2022). We choose to separate these effects due to their distinct dynamical origins; the mean Lagrangian drift is fundamentally set by the vorticity (or lack thereof), whereas the Lagrangian-mean water level is constrained by the geometry of material curves and would be present even in the absence of the mean Lagrangian drift, such as in the rotational Gerstner (1802) wave.

The full solution to (4.27) can be found in Appendix A, although its general character is determined solely by considering the pseudomomentum forcing term. To leading order, this forcing is negative and concentrated near the surface. Assuming the wave packet has a finite width, so that the streamlines must be closed, a clockwise circulation will develop which moves with the packet. This circulation presents itself as a strong jet near the surface, as determined in (4.9) as the classical mean Lagrangian drift, but also includes a slow deep return flow in a direction opposite to that of wave propagation that is well known in the literature (Longuet-Higgins & Stewart 1962; McIntyre 1981; Salmon 2020; Pizzo & Wagner 2025). These studies, however, only constrain the lowest-order mean flow response.

At higher orders, additional forcing terms arise that modify the structure of the mean Lagrangian drift. The third-order forcing terms in (4.27) depend on the quantities 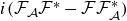 and 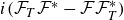 , which represent local fluctuations to the wavenumber and frequency from their characteristic values 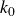 and 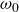 , respectively. These terms therefore account for modifications to the mean Lagrangian drift associated with local variations in the phase speed.

At fourth order, there is a forcing term proportional to the fourth power of the envelope magnitude, which will always act to strengthen the near-surface mean Lagrangian drift, particularly during focusing when its magnitude is most pronounced. The quantity 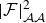 is the curvature of the squared envelope magnitude; it enhances the forcing where the curvature of the envelope is most negative (near the packet centre), and reduces it at the edges where the curvature is positive. Owing to the non-trivial vertical dependence, this effect is reversed at depth. The final contribution, proportional to 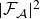 , similarly enhances the forcing near the surface. Together, these fourth-order corrections provide enhancements to the forcing of the mean Lagrangian drift near the surface in regions where wave envelopes are both steep and concave, precisely the conditions present during focusing which led to the greatest observed transport.

Despite the complexity of the full solution, which can be found in Appendix A, the mean Lagrangian drift at the surface can be written explicitly as
(4.28)

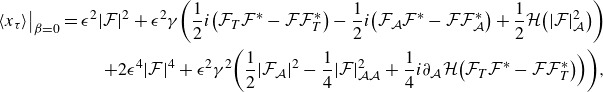

where 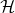 represents the spatial Hilbert transform.

To check that our solutions reduce to known results for steep monochromatic waves, we see what happens when the envelope 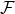 is constant in space. From (4.16), we have
(4.29)

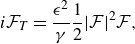

which admits the exact solution (recalling 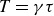 )
(4.30)

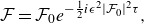

where 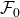 is a complex constant, still 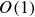 . This slow time modulation to 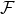 is just the classical Stokes correction to the phase speed for finite amplitude waves (Stokes 1847). Assuming without loss of generality that 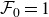 (the physical dimensions can be added later), inserting (4.30) into the trajectories (4.13)–(4.15) yields
(4.31)

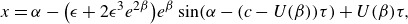



(4.32)

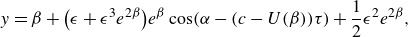



(4.33)

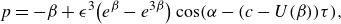




where 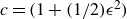 is the nonlinear, non-dimensionalised phase speed, and 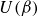 is the mean Lagrangian drift, governed by
(4.34)

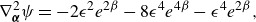

which only depends on 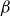 . Therefore, (4.34) reduces to an ordinary differential equation, and by simple integration the solution becomes
(4.35)

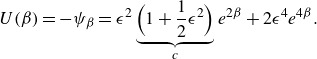




While these are uniformly valid solutions that match those of Clamond (2007), he defines his small steepness parameter 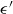 to be equal to 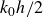 , where 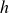 is the crest to trough distance at the surface. The surface crest to trough distance from our solution (4.32) implies the relationship between these small parameters is
(4.36)

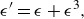

so that using the standard definition of wave steepness 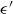 , the drift at the surface can be expressed as
(4.37)

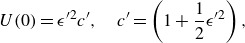

to fourth order in 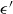 , which matches the results in the literature (Longuet-Higgins 1987). Because unsteady narrow-banded waves do not have an unambiguous geometric reference such as crest to trough height, we must instead settle for its more basic definition above based on the steepness of the first-order Lagrangian expansions.

### 4.2. Comparison with simulation

As a test of the above theory, we directly apply our above expression for the local mean Lagrangian drift of steep, narrow-banded waves (4.28) to estimate the surface transport of focusing wave packets from measurements of the wave envelope, comparing these predictions with the results from our fully nonlinear simulations. Note that the independent variables of the simulation, the initial positions 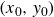 , only coincide with the Lagrangian labels 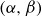 for particles which begin at rest (i.e. where 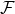 and its derivatives vanish). Since the Lagrangian displacement is only computed for particles which both begin and end at rest, for these particles we may treat both sets of independent variables identically. At the surface the transport is given by integrating (4.28) in time
(4.38)

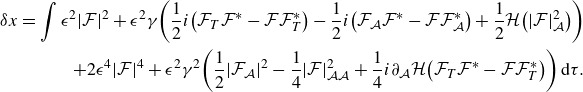




To compare this prediction with the simulations, 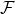 is estimated by using the spatial Hilbert transform of the vertical Lagrangian positions at each time to produce an analytic envelope signal. While this correctly estimates the magnitude of the envelope 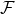 , its phase must be corrected by removing the phase of the carrier wave, which is given by (4.12). Because the theory outlined above is non-dimensional, all quantities must be dimensionalised by a characteristic wavenumber 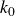 and frequency 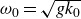 . Note that 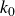 and 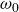 need not be equivalent to the initially specified 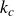 and 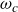 . Because the phase of 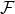 represents narrow-banded deviations from the phase of the carrier wave, we choose 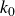 such that these deviations have zero mean when integrated in time. Once the envelope 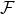 is computed, all that remains is to compute the surface mean Lagrangian transport (4.38) (where 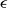 and 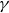 are implicitly included in measurements of 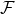 and its slow derivatives) for each particle and integrate in time over the duration of the packet passing to estimate the total transport predicted by this theory. From this, we can compute the maximum and mean transport predicted by narrow-banded theory to compare directly with the simulations.

**Figure 5.**
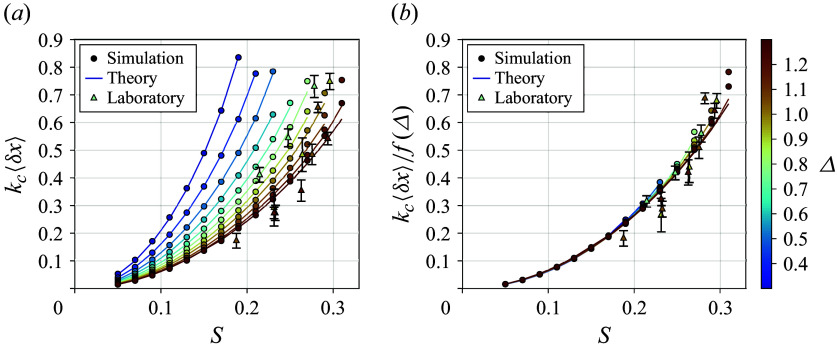
The mean surface transport 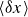 within the focusing region as a function of 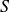 computed both directly from simulation (circles) and using our higher-order theory (4.38) (lines) for each simulation. The mean surface transport for all laboratory experiments (Lenain *et al.* 2019; Sinnis *et al.* 2021) is additionally shown (triangles). In 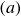 , 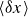 is normalised by the central wavenumber 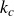 , and each line represents the theoretical prediction of mean transport for each bandwidth value. In 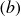 , 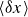 is also normalised by the linear bandwidth dependence 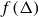 (3.7) which collapses the results. The theory performs best at lower values of 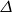 where the narrow-banded envelope assumption is most valid.

Figure 5 presents the mean surface transport obtained directly from the simulations, together with the corresponding theoretical prediction evaluated for each case using (4.38). Also included is the mean surface transport from all available laboratory experiments (Lenain *et al.* 2019; Sinnis *et al.* 2021). In figure 5(*a*), the mean transport is scaled only by the central wavenumber 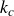 , and good agreement between simulation and theory is found, especially for lower values of 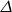 and 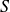 . This is expected since (4.38) is valid to fourth order in the small parameters 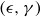 which are proportional to 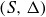 respectively. The theory still performs well across a wide range of parameter space, indicating that the narrow-banded wave approximation is able to capture the local enhancements to the mean Lagrangian drift. Figure 5(*b*) collapses these results, normalising by both 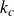 and the linear bandwidth dependence 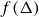 , showing that the bandwidth dependence of the surface transport, even in steep packets, is still reasonably approximated by linear theory. This collapse demonstrates good qualitative agreement between the simulations, theory and experiments, even though neither the theory nor the simulations account for finite-depth effects.

## 5. Discussion

In this paper we investigated the mean Lagrangian drift of two-dimensional irrotational steep focusing surface gravity waves. By working directly in the Lagrangian reference frame, we derived a novel exact technique for constraining the mean Lagrangian drift in general wavy flows, illustrating its role as a spatially varying dynamic mean flow instead of as a passive byproduct of waves. Through a combination of numerical simulations and archived laboratory data, we showed that the surface Lagrangian transport in steep focusing waves can vary spatially and is significantly increased in regions of wave focusing. By performing a separation of scales analysis in the Lagrangian reference frame, we derived Lagrangian particle trajectories in deep-water narrow-banded wave fields, and derived a higher-order expression for the local mean Lagrangian drift and corresponding deep recirculation flow. The form of this expression suggests that wave focusing locally increases the surface drift. Comparing the predictions of this theory with the simulated results shows that it captures a large portion of the observed enhancements especially at smaller bandwidths where the narrow-banded assumption holds.

This study in general advocates for a more local interpretation of the mean Lagrangian drift. For irrotational flow, we showed that in the Lagrangian frame the curl of the mean Lagrangian drift is exactly equal to the curl of the mean pseudomomentum, which itself originates from correlations of wavy particle displacements. Consequently, spatial modifications to the wave orbital motion generate local variations in the mean pseudomomentum, which in turn drive corresponding variations in the mean Lagrangian drift. As evidenced by the particle trajectories within focusing wave packets, this has a profound affect on the way particles are transported – those directly in the focusing region are transported in a rapid burst, whereas particles further downstream drift more slowly as the packet disperses. Modelling the mean Lagrangian drift as a dynamic mean flow which can vary in both space and time may better explain various processes such the enhanced horizontal diffusion due to waves (Herterich & Hasselmann 1982) and could provide more insight into how this mean flow interacts with the vorticity field to generate Langmuir circulation.

These results show that it is the local steepness of the wave field, not just the steepness of individual wave components, which sets the magnitude of these enhancements. That is, even if individual waves comprising a wave field are otherwise well described by linear theory, linear dispersion will consistently create localised focusing events that produce bursts of an increased near surface mean Lagrangian drift. While the likelihood of all wave components constructively interfering such as in the packets studied above may be low, the local steepness need only approach moderate values to begin to see these enhancements, which can occur so long as only some waves constructively interfere. In the real ocean, wave directionality will impact the spatial and temporal scales of both linear and nonlinear wave focusing (Fedele *et al.* 2016; McAllister & van den Bremer 2019), and extending this approach to three-dimensional wave packets is a natural direction for future work. Such focusing events in both two and three dimensions should be commonplace in moderately developed seas, suggesting that both the magnitude and vertical dependence of the mean Lagrangian drift may be incorrectly estimated with models that ignore these effects.

## Appendix A. Streamfunction for the mean Lagrangian drift

Here, we solve Poisson’s equation for the streamfunction of the mean Lagrangian drift (4.27) and compute its value at the surface. In § 4, we showed, that after averaging over the fast orbital motion, the mean Lagrangian drift is divergence free in label space to fourth order, allowing for the introduction of a streamfunction for the mean flow with the following sign convention:

(A1)

Using the exact equivalence between the curl of the mean Lagrangian drift and the curl of the mean pseudomomentum (2.18), this streamfunction was found to obey

(A2)

within the fluid domain, which in label space is just the lower half-plane. The system is closed with boundary conditions  at the surface  and at infinite depth  . The above (A2) is linear, meaning that we can separate  into a homogeneous and particular solution

(A3)

The right-hand side of (A2) only varies slowly in  , so by inspection, the particular solution valid to fourth order is

(A4)

From (A3), we see that  must therefore obey

(A5)

where for brevity  is defined as the right-hand side of (A4) evaluated at the surface. Since the homogeneous system (A5) only depends on the slow variables  and  , we introduce a slow vertical variable  to make explicit that  varies slowly with depth. The homogeneous solution still obeys the Laplace equation in these slow variables (i.e.  , and because the domain is the lower half plane, we can use the theory of Poisson kernels to immediately write the full solution

(A6)

Looking at the form of these solutions, it is clear that  describes the majority of the mean Lagrangian drift near the surface, manifesting as a jet beneath the wave envelope as expected. However, the streamlines of  intersect the surface for a compact packet, which violates the kinematic boundary condition that surface particles remain at the surface. This is remedied by  , which enforces the boundary condition and describes a deep recirculation flow opposite in direction to packet propagation, generating downwelling and upwelling beneath the front and back of the packet, respectively. Combined, the streamlines of  are all closed beneath the surface, indicative of one half of a Bretherton dipole flow (Bretherton 1969). Finally, we note that in a box which contains the entire wave packet, such that  on all boundaries, it is easy to show through the divergence theorem that even for this higher-order solution the total integrated momentum vanishes, which is a well-established result in the literature (McIntyre 1981; Pizzo & Wagner 2025).

From the definition of the streamfunction (A1), we can explicitly derive the mean Lagrangian drift everywhere to fourth order. For the horizontal mean flow this yields

(A7)

where the integral term comes from  . While this integral in general cannot be evaluated in closed form, we can determine its value at the free surface  . Because  obeys Laplace’s equation, it must be the imaginary part of an analytic complex potential  . Along the real axis (  ),  and  are related by the Hilbert transform  so that

(A8)

This, combined with one half of the Cauchy–Riemann equations

(A9)

yields

(A10)

Thus, at the free surface, we can simplify (A7) as

(A11)

where we have used the fact that  as a linear operator commutes with partial derivatives to simplify various terms.

## Appendix B. Fourth-order mean Lagrangian drift for two waves

It is natural to ask whether or not an enhanced mean Lagrangian drift can be found from the simplest wave interaction case, that of two irrotational waves with arbitrary wavenumbers  and  travelling in the same direction. This system is well studied, with nonlinear wave–wave interactions inducing finite amplitude phase speed corrections to each wave while leaving the wave amplitudes time independent (Longuet-Higgins & Phillips 1962). Without loss of generality we set  to ensure that solutions decay with depth. Using  and  to represent the small steepness parameters of each wave, solutions valid to second order in each small parameter are given in Pierson (1961)

(B1)

(B2)

(B3)

where to this order  ,  , with phases

(B4)

where  and  . As we saw previously for linear theory, the mean Lagrangian drift to this order is simply additive for each wave, and we have

(B5)

Solutions to second order can only constrain the Lagrangian pseudomomentum to third order, which has no mean terms. Therefore, we must extend these results to third order in  and  to constrain the pseudomomentum, and as a result the mean Lagrangian drift, to fourth order. Similar to what is necessary for monochromatic waves (Clamond 2007), this requires Doppler shifting the phase by the total mean Lagrangian drift as well as allowing for higher-order corrections to the linear phase speeds. The modified phase can be expressed as

(B6)

(B7)

where  ,  ,  and  are to-be-determined corrections (there are no phase speed corrections proportional to  , Longuet-Higgins & Phillips 1962). From the second-order solutions (B1)–(B3), third-order terms are added of the form, e.g. for  ,

(B8)

for non-negative integers  . Similar expansions are made for  and  . The third-order expansions, using the expanded phases (B6)–(B7), are then inserted into the Euler (2.7)–(2.8), irrotational condition (2.10) and Jacobian gauge (  ) (2.5). Terms proportional to the same powers of  and  are then matched. Because of our phase expansion, all third-order terms are non-secular and can be found by assuming sinusoidal  dependence and exponential  dependence. In the process the corrections to the phase speeds are also constrained. These corrections were found to be

(B9)

(B10)

which match exactly with the results of Longuet-Higgins & Phillips (1962) derived by a purely Eulerian approach. In addition to the standard finite amplitude Stokes correction to the phase speeds, there also exist corrections due to tertiary nonlinear interactions. Notably, these corrections are not symmetric; the relative increase of  from wave 2 depends on the ratio of  whereas the relative increase of  from wave 1 solely depends on wave 1.

From these third-order solutions, including corrections to the phase speeds, we can constrain the fourth-order mean Lagrangian drift  from the mean pseudomomentum at each vertical level. This yields

(B11)

where  and  include nonlinear corrections (B9)–(B10). Reassuringly, the ‘monochromatic terms’ for each wave (i.e. those not multiplied by  ) exactly match the results found above for purely monochromatic waves (4.35). However, there are also interesting interaction terms which are best interpreted through certain limiting examples.

In the case where  , such that we are considering the dynamics of a short wavelength wave riding atop a long wavelength wave, we see from (B9) that the modification to the phase speed of the long wave from the short wave is negligible. However, the phase speed of the short wave is modified by the long wave, precisely by the surface mean Lagrangian drift of the long wave, indicating that the short wave is mainly advected by the surface mean Lagrangian drift of the long wave, which varies much more slowly with depth and as such acts as a constant external current. The mean Lagrangian drift for this limit can be written

(B12)

where, interestingly, the interaction terms not connected to shifts in the phase speed vanish in this limit, which matches very well with the idea that nonlinear wave–wave interactions are strongest between waves of similar wavenumbers (Hasselmann 1962). Therefore, the total mean Lagrangian drift in this long–short wave system can be expressed as a sum of an unmodified long wave drift and a short wave drift whose phase speed is Doppler shifted by the surface drift of the long wave.

Next we can consider the limit where  , where  is small and positive, so that the wave field appears as a series of groups. To lowest order,  and the Doppler shift of each wave is symmetric, with the drift given by

(B13)

Here, we see that, in addition to the standard fourth-order monochromatic drift of each wave, there is a fourth-order interaction term which is positive near the surface, zero at  , and negative below, analogous to what was found for wave packets via a narrow-banded approach in § 4.

In both of these limits, the two waves interact to produce fourth-order positive corrections to the near surface mean Lagrangian drift, which match the results presented in this paper. However, given the analytical difficulty of deriving a higher-order mean Lagrangian drift for even this relatively simple system, it is unlikely that such a direct approach will prove useful for more complex wave configurations.
